# Respiratory rhythm affects recalibration of body ownership

**DOI:** 10.1038/s41598-023-28158-2

**Published:** 2023-01-17

**Authors:** Miku Kosuge, Motoyasu Honma, Yuri Masaoka, Shota Kosuge, Momoka Nakayama, Shotaro Kamijo, Yusuke Shikama, Masahiko Izumizaki

**Affiliations:** 1 Department of Physiology, Showa University School of Medicine, 1-5-8 Hatanodai, Shinagawa-Ku, Tokyo, 142-8555 Japan; 2 Dentsu Lab Tokyo, Dentsu Inc., Tokyo, Japan; 3 Department of Pharmacology, Toxicology and Therapeutics, Division of Physiology, Showa University School of Pharmacy, Tokyo, Japan; 4 Department of Respiratory Medicine, Showa University Fujigaoka Hospital, Yokohama, Japan

**Keywords:** Human behaviour, Respiration

## Abstract

Change in body perception requires recalibration of various sensory inputs. However, it is less known how information other than sensations relates to the recalibration of body perception. Here, we focused on the relationship between respiration and cognition and investigated whether respiratory rhythms are related to the recalibration of hand perception. We built a visual feedback environment, in which a mannequin hand moved in conjunction with its own respiratory rhythm, and participants performed an experiment under conditions in congruency/incongruency for spatial and temporal factors. The temporal and spatial congruency between own respiratory rhythm and the mannequin hand markedly facilitated the phenomenon of hand ownership sense transfer to the mannequin hand, while incongruency had little effect on the change in hand ownership. The finding suggests that an internal model in the brain allows respiratory rhythms to be involved in the adaptation of the body’s neural representations.

## Introduction

Body perception is essential for establishing self-consciousness. Through everyday experience, internal models of the brain constrain the interpretation of sensory input in forming coherent perceptions of the body’s location and ownership^1,2^. Throughout life, the model evolves in response to physical changes owing to biological events, such as limb deficiencies^3,4^ and pregnancy^5^. In shorter time scales, the body’s neural representations strategically adapt to changing behavioral demands, such as during tool use^6^. In the classical rubber hand illusion (RHI), an artificial hand is touched synchronously and tactile stimuli of one’s own hand are perceived as if the artificial hand were a part of one’s body when the observer’s hand is not in view^7^. The RHI causes a recalibration of the spatial location of visual and somatosensory cues, resulting in a distorted perception of the spatial location of the observer’s hand and imbues a sense of ownership to the rubber hand^8^. Multisensory recalibration is a common process that updates the relationships among visual, auditory, and somatosensory signals and adjusts spatial or temporal offsets between senses^9–11^. However, the question that arises is the following: is sensation the only information needed to recalibrate body perception? Given findings, such as the brain-gut correlation^12^, it is possible that information from internal organ may also be involved in higher brain processing, and we need to broaden our perspective and examine information other than sensation.

One candidate information that might contribute to the recalibration of body perception is respiration. Although the most important function of respiration is gas exchange, it is also known to influence cognitive function. For example, it has been reported that manipulation of respiration can increase memory and cognitive performance^13–15^. In contrast, the depth and rhythm of respiration are altered by various factors, such as stress^16,17^. Thus, respiration and cognition influence each other, and their interrelationship in an anatomical and physiological context in terms of the relationship between neuronal oscillations in respiration and brain functions has been proposed^18,19^.

Previous works have reported that respiration modulates self-awareness by using a virtual body flashing synchronously with their respiratory rhythm^20–24^. However, it remains unknown whether this method can transfer body perception in real space to other objects that exist in real space as well. The projection of self-consciousness onto a virtual object and onto a real object have different meanings, and the real object is likely to be rejected (e.g., discomfort with prosthetic hands)^3,4^. By confirming the transfer of body perception to a real object rather than a virtual one, we will be able to examine the recalibration of body perception through the synchronization of vision and respiratory rhythm in accordance with real-life situations, and a clinical application related to the elimination of discomfort with prosthetic hands will also be able to be elaborated.

This study extends previous findings on the contribution of respiratory rhythm to a recalibration of body perception in healthy individuals. We examined that respiratory rhythm is involved in the establishment of body perception, in location sense and ownership sense. Specifically, we proposed that hand perception is recalibrated by the temporal and spatial integration of vision and respiratory rhythms without somatosensory input. Here, we built a visual feedback environment of respiratory rhythm and successfully changed ownership sense for hand in the classical RHI by spatially and temporally manipulating the environment.

## Results

**Figure 1:**
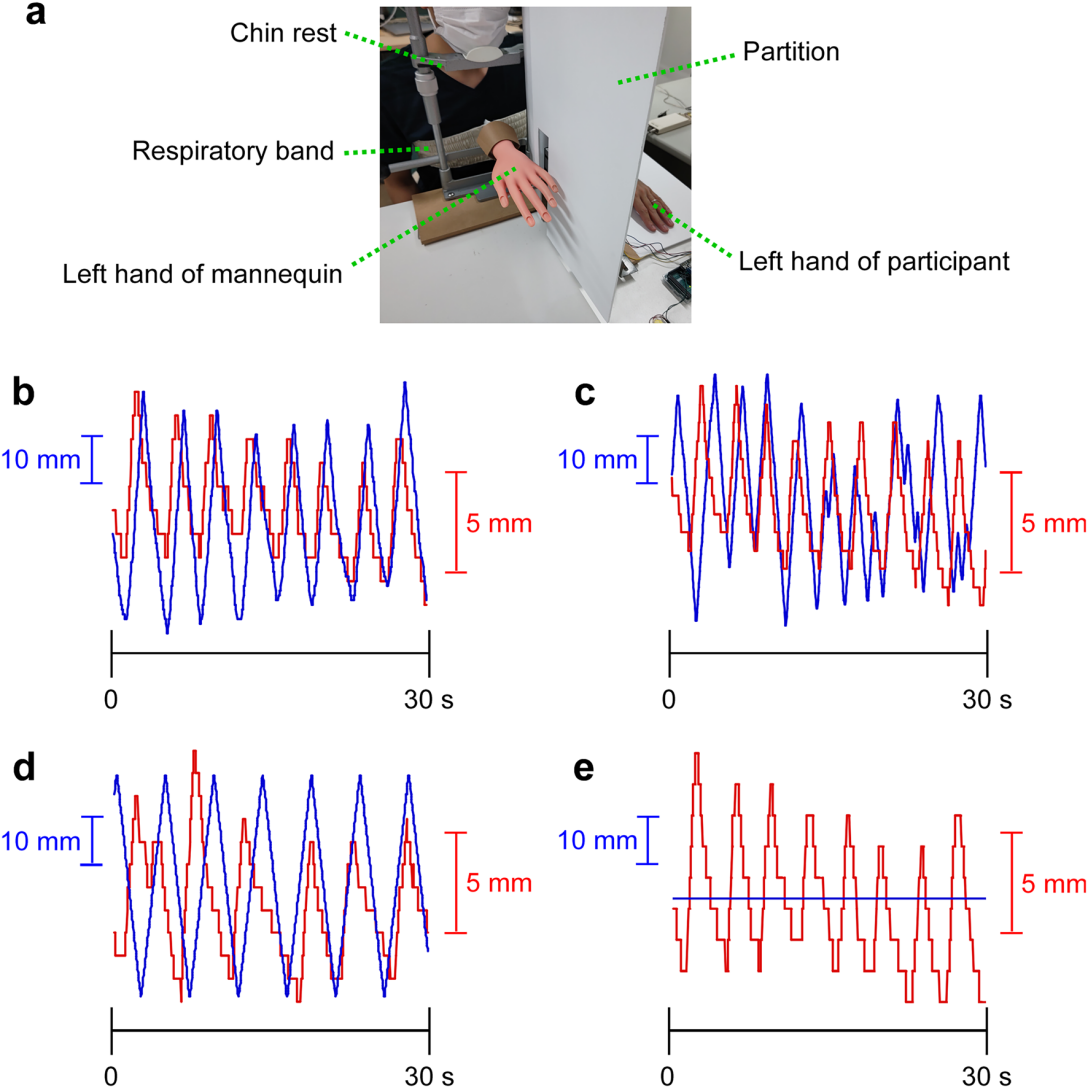
Experimental procedure. (**a**) Experimental device. The mannequin hand moved up and down in real-time in conjunction with the respiratory rhythm. (**b**–**e**), Representative movements of the mannequin hand and respiratory band. The graphs with 30 s extracted from a 2-min trial. Blue lines indicate vertical movements of mannequin hand. Red lines indicate elastic movement of respiratory band. (**b**) Temporal synchronization condition. (**c**) Temporal asynchronization condition. (**d**) Constant speed condition. (**e**) Static condition.

To examine the effect of visual feedback of respiratory rhythm on hand perception in the RHI, we built a device, in which a mannequin hand moves up and down in real-time according to the participant’s respiratory rhythm (Fig. 1a, Supplementary Figs. 1 and 2). We set up four conditions for temporal factors: synchronization condition (Fig. 1b, Supplementary Video 1), asynchrony condition (Fig. 1c, Supplementary Video 2), constant condition (Fig. 1d, Supplementary Video 3), and static condition (Fig. 1e, Supplementary Video 4). For spatial factors, we set up a spatial congruent condition, in which the mannequin and participant’s hands were facing the same direction, and a spatial incongruent condition, in which the fingertips of the mannequin hand were facing the participant and the palm of the hand was facing up (Supplementary Videos 5–8). For each of body ownership and location sense, we calculated the difference in scores between pre- and post- RHI training (2 min) as the illusory amount.

**Figure 2:**
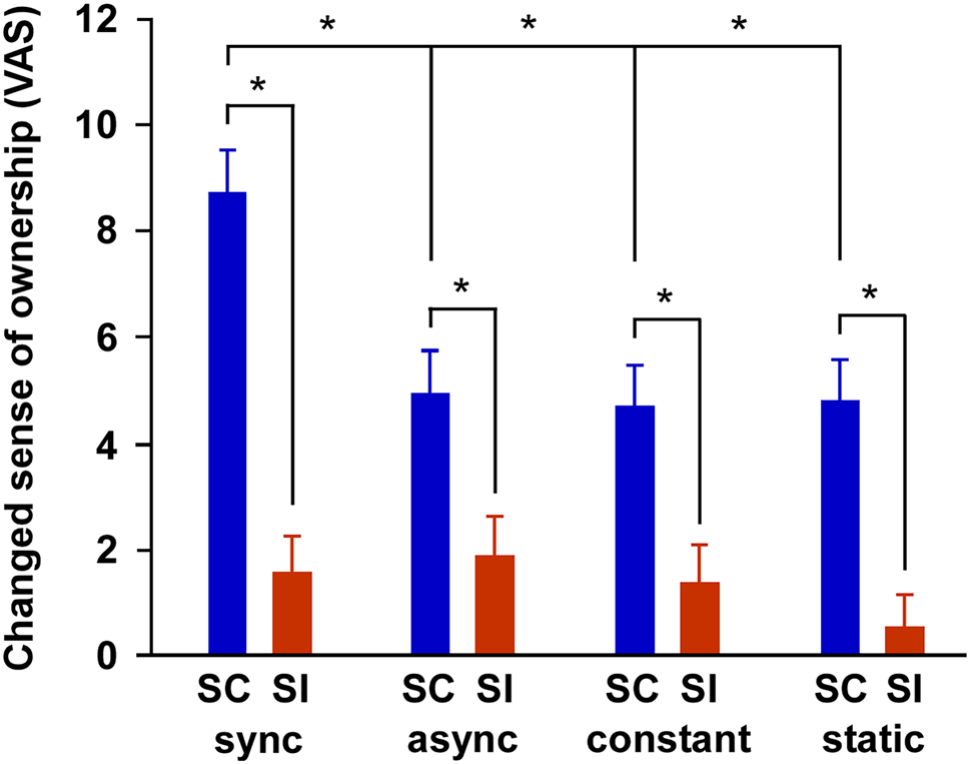
Temporal and spatial congruencies markedly increased a changed sense of ownership. The results of ownership sense. All spatial congruency (SC) conditions increased the changed sense of ownership compared to spatial incongruency (SI) conditions (*p < 0.05). Synchronization condition (sync) increased the changed sense of ownership compared to the conditions of asynchronization (async), constant speed (constant), and static (*p < 0.05). Error bars show the standard error of the mean.

Shapiro–Wilk test showed no significant difference in ownership (*df* = 304, *p* = 0.20) and location sense (*df* = 304, *p* = 0.51). Levene test showed no significant difference in ownership (*df* = 7, 296, *p* = 0.06) and location sense (*df* = 7, 296, *p* = 0.10). Two-way ANOVA showed that there were main effects of temporal factor (*F*(3, 296) = 2.735, *p* < 0.05, *η*^2^ = 0.027) and spatial factor (*F*(1, 296) = 35.622, *p* < 0.0001, *η*^2^ = 0.107) on a changed sense of hand ownership, while there was no main effect of interaction (*F*(1, 296) = 1.754, *p* = 0.156, *η*^2^ = 0.017) (Fig. 2). Post-hoc t-tests revealed that the changed sense of hand ownership in the synchronous condition was greater than that in the asynchronous [*p* = 0.044; 95% Confidence Interval (95% CI) 0.062–7.675], constant (*p* = 0.024; 95% CI 0.352–7.964), and static conditions (*p* = 0.020; 95% CI 0.431–8.043) in the spatial congruency condition. The t-tests also revealed that the changed sense of hand ownership in the spatial congruent condition was greater than that in the spatial incongruent condition in the synchronous (*p* < 0.0001; 95% CI 4.206–9.846), asynchronous (*p* = 0.034; 95% CI 0.233–5.873), constant (*p* = 0.039; 95% CI 0.154–5.794), and static conditions (*p* = 0.005; 95% CI 1.233–6.873). In contrast, the ANOVA showed that there were no main effects of temporal factors (*F*(3, 296) = 0.863, *p* = 0.460, *η*^2^ = 0.009), spatial factors (*F*(1, 296) = 0.206, *p* = 0.650, *η*^2^ = 0.001), and interaction (*F*(1, 296) = 1.496, *p* = 0.216, *η*^2^ = 0.015) on a changed sense of hand location (Supplementary Fig. 3).

## Discussion

We showed that recalibration of hand ownership in the classical RHI is enhanced by satisfying the spatial similarity and temporal synchrony conditions between the respiratory rhythm and mannequin hand. This suggests that hand ownership transfers to a real object by the integration of visual information and respiratory rhythm without somatosensory input.

A previous study reported that the RHI is less likely to occur when there is temporal asynchrony between the visual stimulus of rubbing the mannequin hand with a brush and the tactile stimulus of rubbing one’s own hand with a brush^25^. This indicates that temporal coincidence of somatosensory and visual information is important for recalibration of body perception. The present study showed that the changed ownership in RHI increased more in the synchronization condition than in asynchrony and constant conditions, indicating that continuous temporal coincidence between respiratory rhythm and mannequin hand movement is conclusive clue, rather than simply a moving mannequin hand facilitating the illusion. A physiological study suggests that the insular cortex plays a role in the detection of temporal synchronization between senses^26^. Furthermore, a study showed that attention to respiration increased respiratory rate coherence in the insula^27^. The insula is strongly connected to the amygdala and orbitofrontal cortex (OFC), and is believed to play an important role in body perception^28,29^. The temporal synchrony of respiratory rhythm and mannequin hand movements on the recalibration of body perception may also be related to insular function. In contrast, the difference between spatial congruent and incongruent conditions in ownership illustrated the importance of spatial congruence in the RHI^30,31^, suggesting that spatial similarity was reflected even in the relationship between respiratory rhythm and body perception.

Some studies have pointed out a link between neuronal oscillations and cognitive function. The piriform cortex oscillation correlates with respiratory rhythm^32^. It was also reported that respiration may modulate neuronal oscillations via sensory input from the olfactory bulb^33^. Olfactory bulb neurons generate neural activity by the rhythmic flow of air over the olfactory epithelium, even in the absence of odor^34^, and the piriform cortex has been found to receive direct signals from this region^35,36^. The OFC receives input from the piriform cortex via the amygdala^37^ and the OFC may play a role in linking respiration rhythm and body perception. It is possible that rhythmic signals from the lungs and viscera, which are moved with each breath, influences body perception via these networks. Identifying the region where respiratory rhythm and visual information are integrated is a future challenge.

Why was the effect of respiratory rhythm not reflected in the sense of hand location? The distinction between the ownership and location senses may be partially attributed to the neural dissociation between the functional subsystems underlying each element of the illusion. Ownership generally depends on networks in the frontoparietal cortex that support the different perceptual components of body perception^32^. Activity in the posterior parietal cortex (PPC) is associated with the integration and coordination of multisensory information concerning the body position, whereas activity in the ventral premotor cortex (PMv) is associated with the sense of body ownership^30,38,39^. Transcranial magnetic stimulation of the inferior parietal lobule prior to RHI induction inhibits proprioceptive drift, while changes in the sense of ownership remain unaffected^40^. In contrast, activity in the PMv is correlated with the intensity of the sense of ownership in RHI^39^. Such an independent function may allow for differences in location sense and ownership^41^. Given the relationship between somatosensory input and PPC, the lack of tactile stimulation in the current experiment may have resulted in less PPC involvement and, therefore, the effect of the respiratory rhythm may not have affected location sense. Unlike previous research, which examined the relation between respiratory rhythm and virtual body^20–24^, our result that hand location sense is less likely to cause recalibration than hand ownership sense in the real object, may be a point of caution for application to real hand or body.

In the current results, the effect size of temporal synchronization was low (η^2^ = 0.027). As our RHI experiment dealt with "object in real space," it was expected to be different from the "virtual body," reported in the previous studies^20–24^. Compared to the transference to the virtual body, the transference to real object was weaker, which may have led to the low effect size. In everyday life, ownership transference may be more prone to rejection against physically present objects. At least, the mechanism of transference to virtual body seems to differ from that of transference to real object in the recalibration of bodily perception on the synchronization of vision and respiratory rhythms.

The current study has several limitations. First, the tidal volume of respiratory was not measured in this experiment because the participants could not wear a mask-type respiratory measurement device due to COVID-19. It is possible that the amount of individual respiratory ventilation may have affected the amount of illusion. Second, in the apparatus created in this study, the movement of the mannequin hand was large in relation to the movement of the bust band. This was performed to maximize the experimental effect; however, the experimental effect may have been excessively amplified and may have differed from the usual relationship between vision and respiratory rhythm. The effect of this ratio on the amount of RHI needs to be examined. Third, mannequin hand was used in all experimental conditions, and spatial information for hand was controlled with the forward and reverse hand, as previous studies have reported that a changing the orientation of the mannequin hand decreases the amount of illusion^30,31^. Thus, in this study, no other object was used. It is necessary to examine whether the synchronization effect also occurs for objects without bodily shape and to scrutinize the specific role of respiration in embodiment. Finally, in our experiments, the sense of agency was unmeasured. As sense of agency and ownership are different elements^42^, in the future, agency data should also be obtained and differences from ownership sense should be examined.

The present study shows that respiratory rhythm is constituted to the recalibration of body ownership in classical RHI using a real object. Although we are rarely aware that respiration and body perception are related in everyday life, respiration may be constantly involved in the recalibration of body ownership, including an augmentation of the body ownership through tool use^6^. It is likely that an internal model in the brain allows respiratory rhythms to be involved in the adaptation of the body's neural representations. This study has clinical implications for patients with limb loss who may feel uncomfortable with their prosthetic limbs^4^. The facilitating recalibration of body ownership through respiratory rhythms may reduce their discomfort with the prosthetic limb.

## Methods

### Participants

This study was approved by the ethics committee of Showa University School of Medicine and conducted according to the principles of the Declaration of Helsinki (trial identifier number: 2179). G*Power (Version 3.1.9; University of Dusseldorf, Dusseldorf, Germany) specified that a sample size of 38 would be needed to obtain 85% power to detect a medium effect with an alpha of 0.05. The effect size (0.50) was determined by previous research for RHI^25,43,44^. No data were analyzed prior to collection of the full sample. Thirty-eight university students provided written informed consent prior to the study (mean age, 22.26; range, 19–27; standard deviation, 1.72; 17 female individuals) and participated for 5000 yen. All participants were right-hand dominant and had no history of neurological or psychiatric disease. Participants had normal vision with/without correction.

### Apparatus

We built a device, in which a mannequin hand moved up and down in conjunction with the participant's respiratory rhythm (Fig. 1a, Supplementary Figs. 1 and 2). The respiratory rhythm was measured using a bust band (Respitrace system, A.M.I., Ardsley, NY)^45,46^. The control system was created in the programming language *Processing*, and waveform signals were acquired from the expansion and contraction of the bust band and reflected in the vertical movement of the mannequin hand in real-time (average delay, 0.11 s). The maximum range of movement of the mannequin hand was 10 cm (approximately 2 cm movement of the bust band corresponded to 8 cm of the mannequin hand). The starting point of the mannequin hand was 6 cm above the desk (the center of the range of motion). Each participant sat on a chair, placed the chin on a chin rest, and secured their head with a headband to prevent body movement. The mannequin's left hand was positioned so that it was in front of the participant, and the participant's left middle finger was placed 25 cm to the left of the mannequin's middle finger. Their left hand was unable to be directly observed by the trials. To minimize the influence of environmental noise during the experiment, the participants were asked to wear headphones, and white noise was played. The start of the white noise was used as a cue to begin fixing gaze toward the mannequin hand.

### Experimental conditions

Four conditions were established for the temporal synchronous pattern of the participant's respiration rhythm and the vertical movement of mannequin hand. A temporal synchronization condition was when the respiration rhythm and the mannequin hand movements were synchronized (Fig. 1b, Video 1 in Supplemental Material), an asynchronization condition was when the mannequin hand movement was random and smooth owing to the Perlin noise function (Fig. 1c, Video 2 in Supplemental Material), a constant rhythm condition was when the mannequin hand moved in a fixed rhythm (Fig. 1d, Video 3 in Supplemental Material), and static condition was when that the mannequin hand did not move (Fig. 1e, Video 4 in Supplemental Material). For spatial similarity, two conditions were set: a spatial congruent condition was when the mannequin hand and the mannequin hand were facing the same direction, and a spatial incongruent condition was when the fingertips of the mannequin hand were facing the participant's side and the palm of the hand was facing upward (Videos 5–8 in Supplemental Material). A total of eight conditions were randomly performed per participant.

### Procedures

Participants were instructed to look fixedly at the mannequin hand during the RHI training task, and to make a rating on their location sense and ownership before and after the task^43,44^. Nasal breathing was also instructed, as it was noted that nasal and mouth breathing have different physiological mechanisms^37^. Participants had a 2 min rest period between the tasks, during which the head remained immobilized. The difference between ratings before and after RHI training was analyzed as the amount of illusion. To estimate the location sense, an A2-size blank sheet of paper was pasted on the back of the desk^43,44^. Participants estimated a certain position of the middle finger of their left hand and drew a straight line from the fingertip to the root with their right hand using a pen (Supplementary Fig. 2). The center of the drawn line was judged as the point of location sense, and the distance from the left edge of the desk was calculated. For the ownership rating, participants were asked to draw a vertical line (visual analog scale) on a straight line 10 cm wide to indicate "how much the mannequin hand felt like their own hand" (left side: not at all, right side: strongly feel). The analysis was performed in millimeters.

### Statistics

A normality was checked by the Shapiro–Wilk test and a homoscedasticity was checked by the Levene test. A two-way ANOVA was performed to test the main effects and interactions of temporal factors (synchronization, asynchronization, constant, and static) and spatial factors (congruent and incongruent) on both the values for location sense and ownership. Post-hoc t-tests with Bonferroni correction were performed to test multiple comparisons for variables with significant main or interaction effects. All tests were two-tailed. The results are presented as mean ± standard error of the mean, effect sizes (*η*^2^), and 95% CI SPSS 26.0 (IBM Corp., Armonk, NY) was used for ANOVA.

## Supplementary Information

Supplementary Figures.

Supplementary Video 1.

Supplementary Video 2.

Supplementary Video 3.

Supplementary Video 4.

Supplementary Video 5.

Supplementary Video 6.

Supplementary Video 7.

Supplementary Video 8.

## Data Availability

Raw data from electrophysiological and behavioral data are available from the corresponding authors upon reasonable request.

## Code availability

Custom-made *Processing* codes are available from the corresponding authors upon reasonable request.
