# Supplementary material for: Respiratory rhythm affects recalibration of body ownership
Source: Sci Rep. 2023 Jan 17;13:920. doi: 10.1038/s41598-023-28158-2 (PMC9844178; doi:10.1038/s41598-023-28158-2)
Supplement: Supplementary file 1 — Supplementary Figures. [file 41598_2023_28158_MOESM1_ESM.pdf]

Supplemental Information

M.Kosuge, M.Honma, Y.Masaoka, S.Kosuge, M.Nakayama, S.Kamijo, Y.Shikama, M.Izumizaki

Figures S1–S3

Video descriptions 1–8

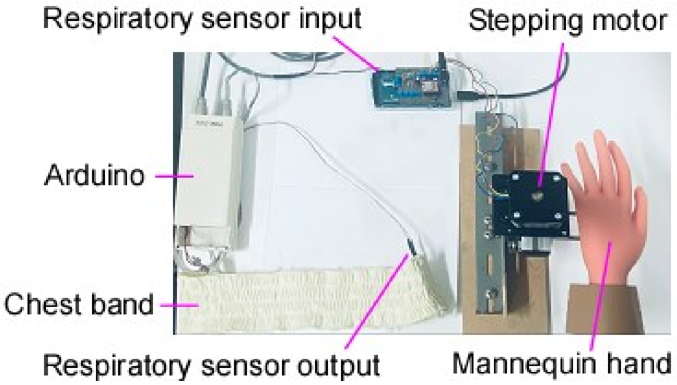

Supplementary Fig 1. Overview of the device. Analog data from the respiration sensor was input to the Arduino, and the respiration data was converted to digital data from 0 to 255. Based on the respiration data, the stepping motor was controlled from a PC via the Arduino.

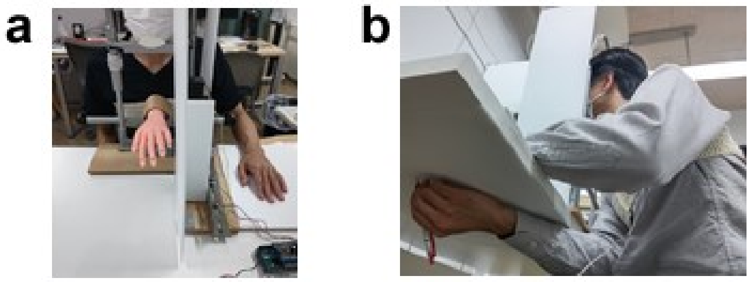

Supplementary Fig 2. Experimental apparatus and procedure. a, The participant was photographed from the front prepared for an experimental trial. **b,** The scene during the estimation of location sense was photographed from below.

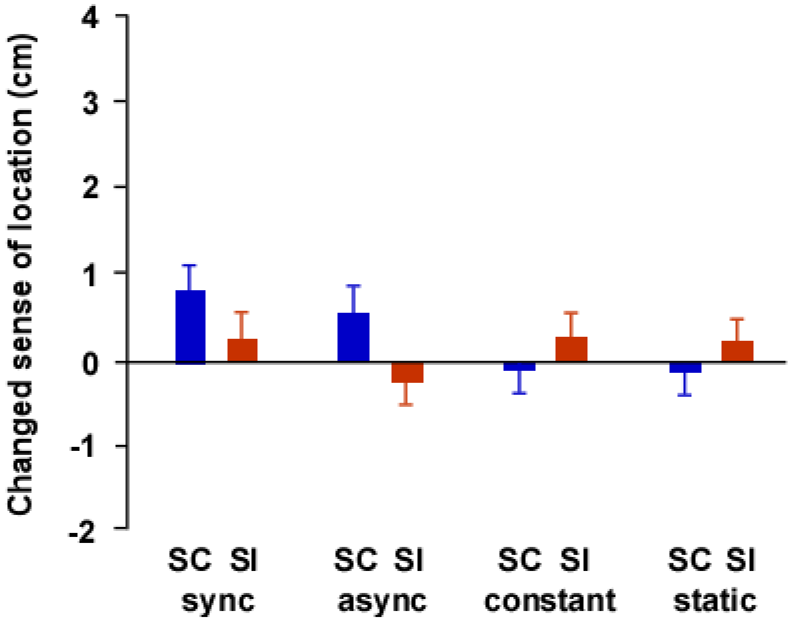

Supplementary Fig 3. The results of location sense. There was no difference in the changed sense of location between spatial congruency (SC) and spatial incongruency (SI) conditions. There was no difference in the changed sense of location among four temporal conditions. Error bars show standard error of the mean.

Video descriptions

Supplementary Video 1. Representative video in the conditions of spatial congruence and temporal synchrony.

Supplementary Video 2. Representative video in the conditions of spatial congruence and temporal asynchrony.

Supplementary Video 3. Representative video in the conditions of spatial congruence and constant speed.

Supplementary Video 4. Representative video in the conditions of spatial concruence and static.

Supplementary Video 5. Representative video in the conditions of spatial incongruence and temporal synchrony.

Supplementary Video 6. Representative video in the conditions of spatial incongruence and temporal asynchrony.

Supplementary Video 7. Representative video in the conditions of spatial incongruence and constant speed.

Supplementary Video 8. Representative video in the conditions of spatial incongruence and static.
